# Scanning Magnetic Microscope Using a Gradiometric Configuration for Characterization of Rock Samples

**DOI:** 10.3390/ma12244154

**Published:** 2019-12-11

**Authors:** Jefferson F. D. F. Araujo, Andre L. A. Reis, Angela A. P. Correa, Elder Yokoyama, Vanderlei C. Oliveira, Leonardo A. F. Mendoza, Marcos A. C. Pacheco, Cleanio Luz-Lima, Amanda F. Santos, Fredy G. Osorio G., Giancarlo E. Brito, Wagner W. R. Araujo, Tahir, Antonio C. Bruno, Tommaso Del Rosso

**Affiliations:** 1 Department of Physics, Pontifical Catholic University of Rio de Janeiro, Rio de Janeiro 22451–900, Brazil; afariassantos@aluno.puc-rio.br (A.F.S.); fosorio@puc-rio.br (F.G.O.G.); tahirjanqau@gmail.com (T.); acbruno@puc-rio.br (A.C.B.); tommaso@puc-rio.br (T.D.R.); 2 Department of Geophysics, National Observatory, Rio de Janeiro 20921-400, Brazil; andrelreis@on.br (A.L.A.R.);; 3 Institute of Geosciences, University of Brasília, Brasília 70910-900, Brazil; angela@erg.inpe.br (A.A.P.C.); eyokoyama@unb.br (E.Y.); 4 Department of Electrical Engineering, State University of Rio de Janeiro, Rio de Janeiro 20550-900, Brazil; mendonza@ele.puc-rio.br; 5 Department of Electrical Engineering, Pontifical Catholic University of Rio de Janeiro, Rio de Janeiro 22451–900, Brazil; marco@ele.puc-rio.br; 6 Department of Physics, Federal University of Piauí, Teresina 64049-550, PI, Brazil; cleanio@ufpi.edu.br; 7 Institute of Physics, University of São Paulo, São Paulo 05508-090, Brazil; gbrito@if.usp.br (G.E.B.); wwlysses@usp.br (W.W.R.A.)

**Keywords:** scanning magnetic microscopy, magnetic measurements, geological sample, equivalent-layer technique

## Abstract

Scanning magnetic microscopy is a tool that has been used to map magnetic fields with good spatial resolution and field sensitivity. This technology has great advantages over other instruments; for example, its operation does not require cryogenic technology, which reduces its operational cost and complexity. Here, we presented a spatial domain technique based on an equivalent layer approach for processing the data set produced by magnetic microscopy. This approach estimated a magnetic moment distribution over a fictitious layer composed by a set of dipoles located below the observation plane. For this purpose, we formulated a linear inverse problem for calculating the magnetic vector and its amplitude. Vector field maps are valuable tools for the magnetic interpretation of samples with a high spatial variability of magnetization. These maps could provide comprehensive information regarding the spatial distribution of magnetic carriers. In addition, this approach might be useful for characterizing isolated areas over samples or investigating the spatial magnetization distribution of bulk samples at the micro and millimeter scales. This technique could be useful for many applications that require samples that need to be mapped without a magnetic field at room temperature, including rock magnetism.

## 1. Introduction

Rock magnetism studies seek to retrieve information regarding primordial magnetic fields in terrestrial and extraterrestrial geological materials by analyzing their remanence magnetizations [1,2]. Even if these materials may hold this magnetic information for millions or even billions of years, the record of the oldest magnetic fields is commonly obliterated by other magnetic records acquired during the geological history of these materials [3]. To recover the complete magnetic history of a geological sample, identifying each magnetic component, traditional techniques estimate magnetization from measurements of the magnetic field outside the sample produced by its remanence. This kind of measurement, i.e., bulk magnetization, reflects the vector sum of the magnetic moments of all magnetic minerals as a function of their volume. Over the past six decades, these techniques have been successful in solving rock magnetic problems that did not require a high degree of detail. However, when the magnetic history is uncertain and complex, e.g., meteoritic magnetizations, the bulk magnetic measurements cannot archive each magnetic component [2].

To overcome these constraints, high precision magnetometer devices have been developed in the last decade [4]. In this sense, a significant advance in rock magnetism studies can be accomplished with the use of scanning magnetic microscopes [5,6]. In spite of this, most scanning magnetic microscopes require a cryogenic system, which greatly increases operating and maintenance costs, making them unviable for most low-cost laboratories. Besides the cost, another problem related to magnetic microscopy lies in the elimination of some ambiguities inherent in this kind of measurements [7]. For these issues, some solutions have been recently developed, such as nonsuperconducting scanning magnetic microscopes and new methods of micromagnetic processing and modeling [7,8,9,10,11,12]. Based on the issues and solutions presented before, this study presented a development of nonsuperconducting scanning magnetic microscopy, which would be easily reproduced and used in low-cost laboratories, as well as in classrooms to teach physics, engineering, geophysics, and geology. The scanning magnetic microscopy was used for the magnetic characterization of millimeter-scale samples in an environment that could be either protected or not by a magnetic shield that achieves a spatial resolution of 200 µm, as shown in the electronic Supplementary Materials (ESI). Using both samples of magnetic microparticles with a small mass (60 µg) and geological samples, the configuration used could measure the remanent magnetization of the samples in the z-direction, i.e., perpendicular to the sample. The device had a scanning range from 150 mm to 150 mm with micrometer resolution mentioned in the ESI. In the current configuration, the microscope was equipped with commercial Hall-effect sensors, as shown in Supplementary Materials Figure S1 in the ESI. The output noise measured at 6 Hz was approximately 520 nT_rms_/√Hz (± 10 nT_rms_/√Hz) in a protected environment, and the magnetic moment sensitivity was 9.20 × 10^−10^ Am^2^ [5,13,14,15]. The standard deviation of the measurements was approximately 0.04 × 10^−10^ Am^2^ (about 0.38%) for the remanent magnetization. A low-cost device capable of filtering and amplifying the signals collected by the Hall-effect sensors, with the same quality as similar equipment offered in the market, was also developed, making it accessible and operational in academic environments [16,17,18]. We tested the device’s performance with magnetic microparticles, containing a core of iron oxide and geological samples.

Maps of a single component of the magnetic field contain information regarding the other two components. For this reason, maps of the *x*- and *y*- components of the magnetic field can be estimated from *z*-component measurements. Vector field maps are valuable tools for the magnetic interpretation of samples with high spatial variability of magnetization. These maps can provide comprehensive information regarding the spatial distribution of magnetic carriers. Moreover, field maps can be useful for characterizing isolated areas over the samples, investigating the spatial magnetization distribution of bulk samples at submillimeter and millimeter scales, and guiding inverse problems for extracting information about magnetic carriers. The amplitude of the magnetic field vector calculated from three estimated components can also show regions devoid of magnetic sources. There are several techniques for estimating the three components of the magnetic field in the Fourier domain using magnetic microscopy data [19]. However, this procedure is commonly used in exploration geophysics for processing total-field anomaly data in the spatial domain by using an equivalent-layer technique [20,21,22]. Here, we showed an application of an equivalent layer for processing magnetic microscopy data in the spatial domain.

## 2. Magnetic Microscope

### 2.1. Mechanical Design and Hall Sensors

**Figure 1:**
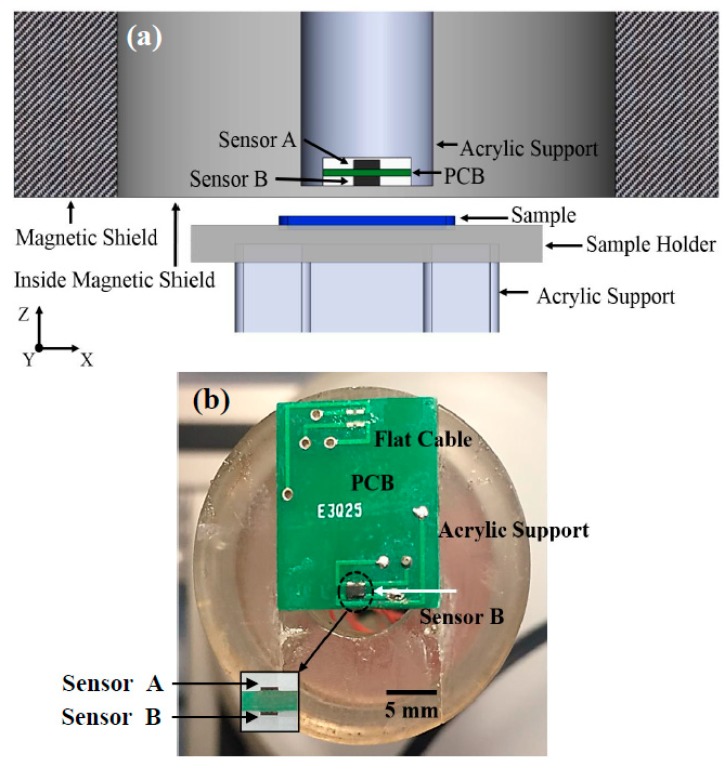
(**a**) Diagram of the main components of the microscope: circuit board containing the gradiometric sensors (A and B) and sample holder, which moves in the X and Y directions. All equipment is inside a magnetic shield. The diagram is not drawn to scale. (**b**) Photo of the Hall sensors coupled in an acrylic structure.

The magnetic microscope was capable of scanning magnetic samples (bulk, liquid, micro- or nanostructured), which were placed in the sample port (Figure 1a and Figure S1 (ESI)). The sample was placed face-up (positive direction, *z*-axis) on the sample holder using adhesive tape (Figure 1a). In order to detect the response generated by the sample, we used two commercial Hall sensors (AKM, Co., San Jose, CA, USA), hereinafter referred to as the Sensor A and Sensor B, which incorporated a GaAs element in surface-mount technology (SMT) package. The sensor detection areas were 200 μm in diameter, and they had a distance of 125 μm (after calibration) to the upper surface. Both sensors were connected in an axial gradiometer configuration and were fixed on the opposite sides of a printed circuit board (Figure 1b). Sensor A was fixed to the circuit board next to the sample with a clear epoxy resin. To approach the sample, sensor A was cut until its 4 connecting terminals appeared on the upper surface. Sensor B acted to reduce the external noise that the shield was unable to eliminate. We also built all the electronics for data acquisition, as shown in the ESI and Figures S2 and S3.

### 2.2. Calibration and Magnetic Measurements

**Figure 2:**
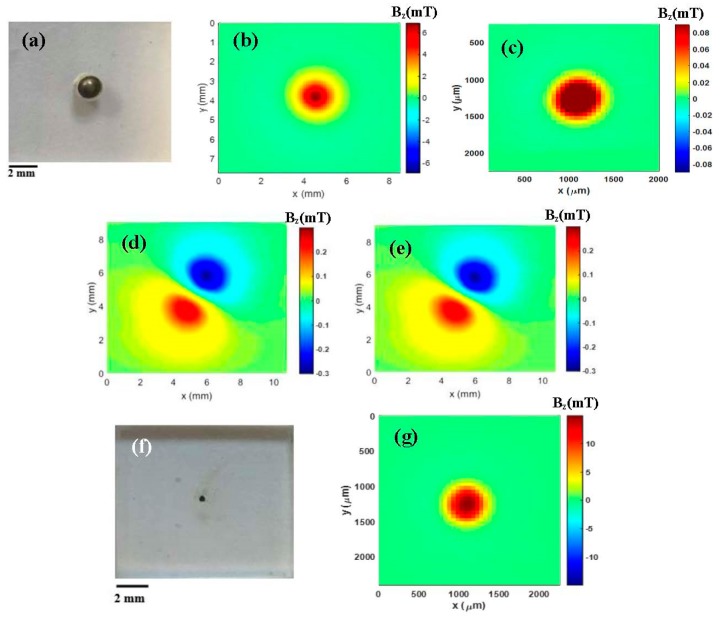
(**a**) Acrylic sample holder with a cylindrical cavity in which the 99% purity nickel sphere was placed. (**b**) Map of the remnant magnetization of the nickel sphere after being magnetized by a 0.5 T magnetic field. (**c**) Map of the remnant magnetization of iron oxide nanoparticles. (**d**) Map of the remnant magnetization of the sphere measured using only circuit boards after a demagnetization process. (**e**) Map of the remnant magnetization of the same sphere measured using the commercial Lock-In amplifier after a demagnetization process. (**f**) A figure representing microparticles of iron oxide. (**g**) Map of the remnant magnetization of iron oxide microparticles.

The calibration process of the magnetic microscope consists of acquiring the distance on the *z*-axis between the sensitivity region of the Hall-effect sensors and the surface of the sample using only circuit boards and measuring the sample remanent fields with a 99% purity nickel sphere magnetized at 0.5 T to determine the distance [13,14,15,17,18]. The nickel sphere was placed in a sample holder made of acrylic material with a cylindrical cavity (see Figure 2a). Figure 2b shows the map of the remanent magnetic field of the nickel sphere. Using the magnetic map and the model of a magnetic dipole, the magnetic moment of the nickel sphere was determined [5,13]. After finding the magnetic moment of the nickel sphere, it was possible to estimate the distance between the sensitivity region of sensor A and the surface of the sample, which was approximately 115 µm. This number was confirmed again using a different sample from the nickel sphere. We performed the same calibration process using magnetic nanoparticles synthesized by pulsed laser ablation (PLA) in liquid (see Figure 2c). We used magnetic nanoparticles with a small mass in the order of tens of µg. This calibration was crucial because it was through this technique that we had the accuracy of the equipment. The results of such procedure had a standard deviation of approximately 0.04 × 10^−10^ Am^2^ (about 0.38%) for the remanent magnetization.

In order to verify the assembly capability, we used only circuit boards and compared the measurements with magnetic maps obtained with commercial equipment (as shown in the ESI), such as the Lock-In amplifier (SR560, SRS Inc.), using the same 99% purity nickel sphere, which was analyzed after being magnetized by a 0.5 T field, and magnetic microparticles of Fe_3_O_4_ obtained by the coprecipitation method [14,18]. Thus, we made scanning magnetic maps of the x- and y-axes (Figure 2b,e,g). Unlike the calibration process, the maps of Figure 2d,e were obtained after the nickel sphere was demagnetized. After this process, the scanning magnetic map was prepared. Figure 2d shows the map obtained using only circuit boards, while the map in Figure 2e shows the map obtained using the commercial Lock-In equipment (SR560, SRS Inc., Sunnyvale, CA, USA). The intensity of the remanent magnetic field in these maps (Figure 2d,e) was lower than that in Figure 2b by order of magnitude due to the demagnetization process. Notably, there was essentially no difference in the magnetic maps of the partially demagnetized spheres. This result was in agreement with the graph in Figure 2b.

We also mapped the remanent field of Fe_3_O_4_ microparticles obtained by the coprecipitation method. Approximately 50 µg of magnetic Fe_3_O_4_ microparticles were placed in a cylindrical cavity in the acrylic sample holder, which had a diameter of 400 µm and a depth of 400 µm (Figure 2f). Figure 2g shows the map of this cylindrical cavity. Through the magnetic map, we could obtain the moment using a model of a cylinder that took the shape of the sample in the cylindrical cavity. In addition, we could also map the remanent fields of magnetic nanoparticles produced by laser ablation that have very small diameters (see Figure 2c). This type of magnetic map and magnetic moment obtained by magnetic microparticle scanning microscopy might be important for a number of applications, such as in vitro and in vivo studies.

## 3. Processing Magnetic Data Using Equivalent Layer Technique

We illustrated in this section how the equivalent-layer technique could be used to estimate the three components and the amplitude of the magnetic field using magnetic microscopy data. We applied this technique for processing the data from two geological samples. Imposing a magnetization direction for the equivalent layer, we estimated a magnetic moment distribution by solving an inverse problem and then calculating the three components of the magnetic field.

### 3.1. Parametrization and Forward Problem

Consider a right-handed Cartesian coordinate system with *z* being oriented positively downward, *x* to the north, and *y* to the east. Let$\;B_{z}^{0}$ be the *N* × 1 vector whose the *i*th element $B_{z}^{i}$ is the *z*-component of the induction magnetic field at the observation point (*x^i^*, *y^i^*, *z^i^*) over a plane above a rock sample. In order to estimate the other magnetic field components, we used a layer composed of *M* dipoles with unit volume, all of them positioned at a constant depth of *z = h*. Mathematically, the predicted *z*-component of the magnetic field produced by the set of dipoles at the point (*x^i^*, *y^i^*, *z^i^*) is given by

(1) $$B_{z}^{i}=\;\sum_{j=i}^{M}m^{i}a_{z}^{ij},$$

where *m^i^* is the magnetic moment of the *j*th dipole and

(2) $$a_{z}^{ij}=\gamma_{m}M_{z}^{ij}\hat{m},$$

in which γ*_m_* is a constant proportional to the vacuum permeability, $M_{z}^{ij}$ is a 3 × 1 vector equal to

(3) $$M_{z}^{ij}=[\partial_{xz}\phi^{ij}\partial_{yz}\phi_{\;}^{ij}\partial_{zz}\phi_{\;}^{ij}]$$

where $\partial_{\lambda z}\phi_{\;}^{ij}$, λ = *x*, *y*, *z*, is the second derivative with respect to the Cartesian coordinates *x^i^*, *y^i^*, and *z^i^* of the scalar function

(4) $$\phi_{\;}^{ij}=\;\frac{1}{\sqrt{{\left(x^{i}-x^{j}\right)}^{2}+{\left(y^{i}-y^{j}\right)}^{2}+{\left(z^{i}-h\right)}^{2}}}$$

in which *x^i^*, y^i^, and *h* are the Cartesian coordinates of the *j*th dipole composing the layer. The $\hat{m}$ is a 3 × 1 unit vector with the magnetization direction of all equivalent sources equal to

(5) $$\hat{m}=\;\left[\begin{array}{c} cos\;I\;\;cos\;D\\ cos\;I\;\;sin\;D\\ \sin I \end{array}\right],$$

where the *I* and *D* are the inclination and declination, respectively. This modeling is solved by using a Python library called Fatiando a Terra [23]. In matrix notation, the predicted *z*-component of the magnetic field produced by the model is given by

(6) $$B_{z}^{p}=\;A_{z}m,$$

in which $B_{z}^{p}$ is an *N*-dimensional vector whose *i*th element is the predicted *z*-component of the magnetic field at the point (*x^i^; y^i^; z^i^*), *A**_z_*** is an *N* × *M* sensitivity matrix whose *ij*th element is defined by the harmonic function $a_{z}^{ij}$ (Equation (2)), and *m* is the *M*-dimensional parameter vector whose *j*th element is the magnetic moment of the *j*th positioned at the point (*x^i^*, *y^i^*, *h*). Moreover, the parameter vector *m* represents the magnetic moment distribution over the layer.

### 3.2. Inverse Problem

The inverse problem consists of estimating the magnetic moment distribution that minimizes the difference between the observed data $B_{z}^{0}$ and the predicted data $B_{z}^{p}$ (Equation (6)). In order to estimate a stable solution m*, we solved the constrained problem of minimizing the goal function

(7) $$Ʈ\left(m\right)=\|B_{z}^{0}-B_{z}^{p}\left(m\right){\|}_{2}^{2}+µ\|m{\|}_{2}^{2}$$

where the first and the second terms of Equation (7) are the data-misfit functions and the zeroth-order Tikhonov regularization function, respectively, µ is the regularizing parameter, and ${\left\|.\right\|}_{2}^{2}$ denotes the squared Euclidian norm. The least-squares estimate of the parameter vector m* is given by

(8) $$m^{*}={\left(A_{z}^{T}A_{z}+µI\right)}^{-1}A_{z}^{T}B_{z}^{0}$$

in which the superscript *T* stands for transposition, and *I* is the identity matrix of order M. After estimating the magnetic moment distribution m*, we could calculate the two other components of the magnetic field using the relations

(9) $$B_{x}^{p}=\;A_{x}m^{*}$$

and

(10) $$B_{y}^{p}=\;A_{y}m^{*}$$

in which $B_{x}^{p}$ and $B_{y}^{p}$ are the N-dimensional predicted vectors of the *x*- and *y*-components of the magnetic field, respectively, and $A_{x}^{p}$ and $A_{y}^{p}$ are *N* × *M* matrices whose *ij*th elements are, respectively, given by

(11) $$a_{x}^{ij}=\gamma_{m}M_{x}^{ij}\hat{m}\;,$$

and

(12) $$a_{y}^{ij}=\gamma_{m}M_{y}^{ij}\hat{m}\;,$$

where

(13) $$M_{x}^{ij}=[\partial_{xx}\phi_{\;}^{ij}\partial_{xy}\phi_{\;}^{ij}\partial_{xz}\phi_{\;}^{ij}{]}^{T}$$

and

(14) $$M_{y}^{ij}=[\partial_{yx}\phi_{\;}^{ij}\partial_{yy}\phi_{\;}^{ij}\partial_{yz}\phi_{\;}^{ij}{]}^{T}$$

in which $\partial_{\lambda z}\phi_{\;}^{ij}$, λ = *x*, *y* are the second derivatives of the scalar function $\phi_{\;}^{ij}$ with respect to the Cartesian coordinates *x^i^*, *y^i^*, and *z^i^* of the observation points, analogous to Equation (2). Finally, we could calculate the amplitude of the magnetic field as follows:

(15) $$B=\sqrt{B_{x}^{p^{2}}+\;B_{y}^{p^{2}}+B_{z}^{p^{2}}}$$

where $B_{x}^{p}$, $B_{y}^{p}$, and $B_{z}^{p}$ are the *x*-, *y*- and *z*-components of the magnetic field, *respectively*, and *B* represents the amplitude.

## 4. Results

To test the efficiency of the present microscope on natural samples, we chose two different geological samples. A short overview of the geological context of these samples is explained as follows. The first one was collected from the Parnaíba basin and the other from the Vredefort crater. In addition to the vertical component maps, we formulated an inverse problem to obtain the other components and the amplitude of the magnetic field vector.

### 4.1. Parnaíba Sample

**Figure 3:**
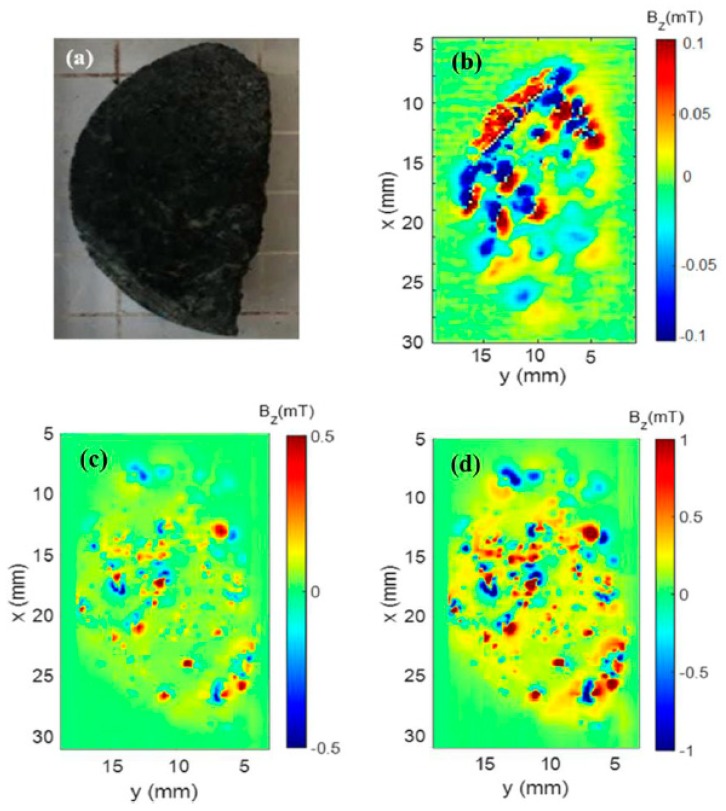
(**a**) Photo representing a sample taken from the Brazilian state of Tocantins. (**b**) Magnetic map of the Parnaíba sample representing the natural remanent magnetization. (**c**) Magnetic map of Parnaíba sample after applying 200 mT. (**d**) Magnetic map of Parnaíba sample after applying 400 mT.

In this study, we collected samples from the basaltic dikes of Paraíso do Tocantins City, located at the western border of the Parnaíba Basin, in its local basement. The dikes host rocks were formed by metamorphic rocks from the Araguaia Fold Belt. By geological field control, we estimated that these dikes belong to the Triassic-Jurassic boundary volcanic suite [24]. Continental magmatic events have been recorded on several tectonic provinces at the South America Platform [24]. These events included the formation of dikes, sills, and flows of basaltic rocks that occur in both sedimentary basins and orogenic belts. The Parnaíba Basin is one of the largest cratonic sedimentary basins in South America, with an area of 665,888 km^2^. The basin is bordered to the west by the Tocantins Province and the east by the Borborema Province. The basaltic rocks (Figure 3a) of Parnaíba are related to the opening of the Atlantic Ocean, both at the Triassic-Jurassic boundary and at the Early Cretaceous period.

We presented in Figure 3 the results of the magnetic microscopy measurements of the Parnaíba samples (Figure 3a). In Figure 3b, we presented the microscopy map of the sample with its natural remanence magnetization (NRM). We could observe that, in this map, there were regions with different magnetic field intensities and with opposite directions. This measurement was performed within a magnetic shield, and the maximum magnetic field strength was 0.1 mT. This value implied that this sample might have undergone some secondary magnetization. To verify the behavior of the magnetic minerals of the rock sample, we applied a 200 mT and 400 mT magnetic field in the *z*-axis direction and then mapped the sample in the scanning magnetic microscopy (Figure 3c,d). We could observe in Figure 3c,d that there were changes in intensities and directions of the sample field. These changes could be explained by the variation of the total vector of the sample’s magnetic moment caused by the applied magnetic field.

#### Equivalent Layer Application to the Parnaíba Basin Sample Data

**Figure 4:**
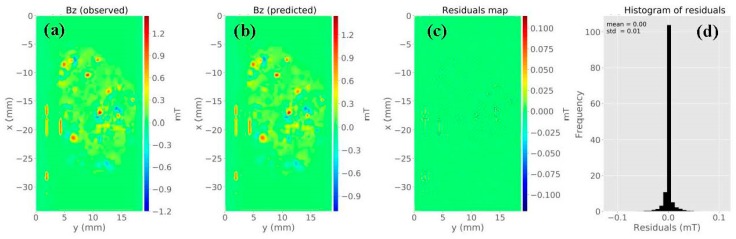
Application of the equivalent-layer technique to microscopy data from the Parnaíba basin sample. (**a**) Observed *z*-component measured by the magnetic microscope. (**b**) Estimated *z*-component produced by the layer. (**c**) Difference between panels (**a,b**). (**d**) Histogram of the residuals.

In order to demonstrate the procedure of processing magnetic data, we applied the equivalent-layer technique in the map shown in Figure 3d. The observed data were measured on a regular grid of 128 × 70 (a total number of *N* = 8960 observations) over an area extending approximately 34 mm and 18 mm along the *x*-axis and *y*-axis, respectively (Figure 4a). The sensor-to-sample distance was 138 µm above the sample surface. We used a layer formed by a grid of 128 × 70 dipoles (a total of *M* = 8960 equivalent sources) positioned at a constant depth of *z* = 633 µm below the observation plane. The magnetization direction for all dipoles was equal to 90° and 0° for inclination and declination, respectively, with the same direction as the imparted field.

**Figure 5:**
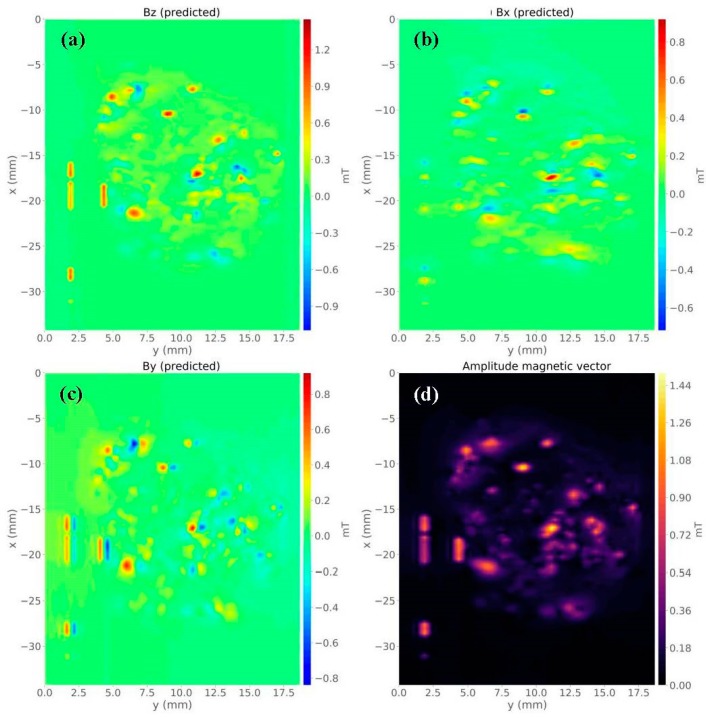
Magnetic vector components of the Parnaíba basin sample calculated from the equivalent-layer technique. (**a**) Map of estimated *z*-component. (**b**) Map of estimated *x*-component. (**c**) Map of the estimated y-component. (**d**) Amplitude calculated from the estimated magnetic field components.

By solving Equation (8) using a regularizing parameter µ = 10^−15^, we estimated a magnetic moment distribution over the layer (not shown). Figure 4b is the predicted data produced by the equivalent layer. Figure 4c shows the residuals map, which is defined as the difference between the observed data (Figure 4a) and the predicted data (Figure 4b). The histogram of residuals appeared with a mean of 0 mT and a standard deviation of 0.01 mT (Figure 4d). From all these results, we could conclude that the estimated magnetic moment distribution produced an acceptable data fitting. Figure 5a–c shows the predicted *z*-, *x*-, and *y*-components of the magnetic field, respectively. We calculated the amplitude of the magnetic field using the estimated three components (Figure 5d). The last result showed the concentration of magnetic carriers and the zones devoid of sources along with the Parnaíba sample. As shown in Figure 3 and Figure 5, we concluded that the equivalent-layer technique could be a useful tool for determining the magnetic vector components and its amplitude.

### 4.2. Vredefort Sample

The impact crater is the fastest known geological process. High shock pressures (>5 GPa) and high shock temperatures (>1000 °C) are responsible for the formation of unique geochemical systems. The evolution of these systems, i.e., the formation of new mineralogy, can generate complex petrophysical signatures [25,26,27]. An example of this kind of signature can be observed on the rock magnetic data of the Vredefort Dome (South Africa) [28]. The Vredefort Dome is the largest impact structure known on Earth, with a diameter of approximately 250 km, and magnetic studies of the dome have been performed since the 1960s [22]. Vredefort has several types of impactites, i.e., impact melt veins, granophyric dikes, shatter cones, etc. In this context, a recurrent target of paleomagnetic studies is the impact melt veins, especially the pseudotachylite veins. Pseudotachylites or pseudotachylite breccia are very fine-grained or glassy rock formed mainly by friction melt. This kind of rock has been reported in many failure and shear zones and some impact structures, such as the Vredefort Dome [29,30].

**Figure 6:**
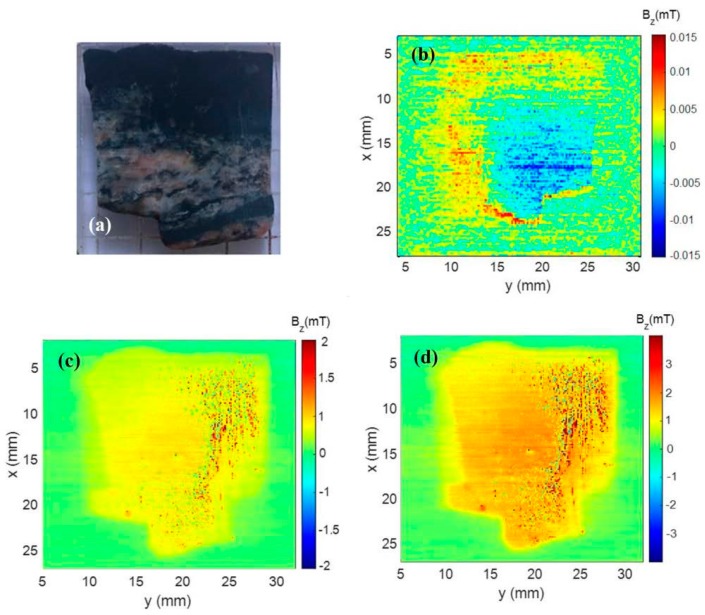
(**a**) Photo of the Vredefort sample. (**b**) Magnetic map of the Vredefort sample representing the natural remanent magnetization. (**c**) Magnetic map of Vredefort sample after applying 200 mT. (**d**) Magnetic map of Vredefort sample after applying 400 mT.

In this study, we used samples of the pseudotachylite veins collected on the Leeukop Quarry at the Vredefort Dome during a 2008 field trip (Figure 6a). These samples were similar to those used for paleomagnetic studies. In Figure 6, the results of the magnetic microscopy measurements of the Vredefort sample are shown (Figure 6a). In Figure 6b, we presented the microscopy map of the sample with its natural remanence magnetization. We could observe that in this map, there were apparently only two regions of different intensities in the sample. This measurement was performed within a magnetic shield with a maximum magnetic field of 15 mT. Although the remanent magnetic field of the Vredefort sample was lower than the sample, it was still high compared to the Earth’s magnetic field. Therefore, this implied that this sample might have undergone some secondary magnetization. To verify the behavior of the magnetic minerals of the rock sample, we applied a 200 mT and 400 mT magnetic field in the *z*-axis direction and then mapped the sample in the scanning magnetic microscopy (Figure 6c,d).

#### Equivalent Layer Application to the Vredefort Sample Data

In order to demonstrate the procedure of processing magnetic data, we also applied the equivalent-layer technique in the map shown in Figure 6d. The observed data were measured on a regular grid of 121 × 99 (a total number of *N* = 11,979 observations) over an area extending 36 mm and 30 mm along the *x*-axis and *y*-axis, respectively (Figure 6a). The sensor-to-sample distance was 138 µm. We used a layer formed by a grid of 121 × 99 dipoles (a total of *M* = 11,979 equivalent sources) positioned at a constant depth of *z* = 818 µm below the observation plane. The magnetization direction for all dipoles was equal to 90° and 0° for inclination and declination, respectively, with the same direction as the imparted field.

**Figure 7:**
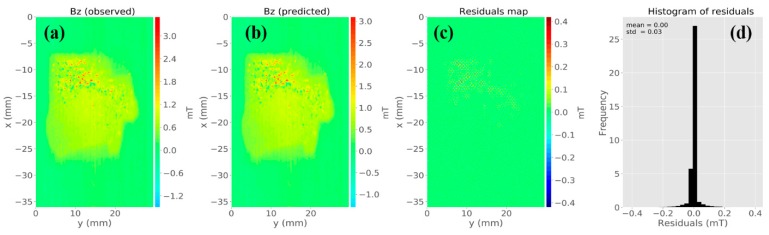
Application of the equivalent-layer technique to microscopy data from the Vredefort sample. (**a**) Observed *z*-component measured by the magnetic microscope. (**b**) Estimated *z*-component produced by the layer. (**c**) Difference between panels (**a**,**b**). (**d**) Histogram of the residuals.

**Figure 8:**
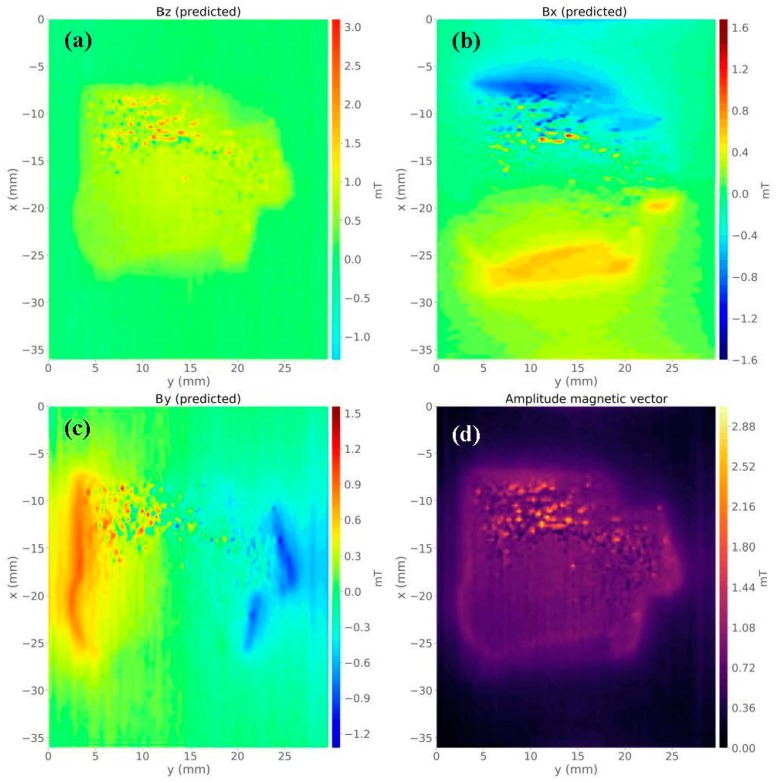
Magnetic vector components of the Vredefort sample calculated from the equivalent-layer technique. (**a**) Map of estimated *z*-component. (**b**) Map of estimated *x*-component. (**c**) Map of estimated *y*-component. (**d**) Amplitude calculated from the estimated magnetic field components.

By solving Equation (8) using a regularizing parameter µ = 10^−15^, we estimated a magnetic moment distribution over the layer (not shown). Figure 7b is the predicted data produced by the equivalent layer. Figure 7c shows the residuals map, which is defined as the difference between the observed data (Figure 7a) and the predicted data (Figure 7b). The histogram of residuals appeared with a mean of 0 mT and a standard deviation of 0.002 mT. It means that the estimated magnetic moment distribution produced an acceptable data fitting. Figure 8a–c shows the predicted *z*-, *x*-, and *y*-components of the magnetic field, respectively. We calculated the amplitude of the magnetic field using the estimated three components (Figure 8d). The result in Figure 8d shows a concentration of magnetic carriers on the upper bound of the Vredefort sample. We could conclude after results shown in Figure 7 and Figure 8 that the equivalent-layer technique could be a useful tool for determining the magnetic vector and its amplitude.

## 5. Conclusions

Thin and polished sections of the geological samples were successfully scanned using a scanning magnetic microscope. After analyzing the magnetic field images of the geological samples, a variation in the magnetic field intensity was observed. Moreover, we applied the equivalent-layer technique to process the vertical component of the magnetic field generated by the Parnaíba Basin sample and the Vredefort sample. Different from the Fourier domain approach, we calculated the three components and the amplitude of the magnetic field formulating an inverse problem in the spatial domain. This technique could be a useful tool to describe the magnetization distribution over the sample, identifying regions with or without magnetic carriers along with geological samples.

## Supplementary Materials

The following are available online at https://www.mdpi.com/1996-1944/12/24/4154/s1, Figure S1. Image of the Hall microscope and its components; Figure S2. (a) Circuit consisting of current sources and instrumentation amplifiers for the amplification. (b) Noise analysis graph between the Lock-In AJE equipment and the commercial Lock-In equipment. (c) Noise analysis graph of the gradiometer system with and without the protection of the magnetic shield. Figure S3. Electric circuit of a Lock-In amplifier using the AD620, AD630, and OP27 integrated circuits.
