# Supplementary material for: Scanning Magnetic Microscope Using a Gradiometric Configuration for Characterization of Rock Samples
Source: Materials (Basel). 2019 Dec 11;12(24):4154. doi: 10.3390/ma12244154 (PMC6947304; doi:10.3390/ma12244154)
Supplement: Supplementary file 1 [file materials-12-04154-s001.pdf]

*materials*

MDPI

*Article*

# Scanning Magnetic Microscope Using a Gradiometric Configuration for Characterization of Rock Samples

**Jefferson F. D. F. Araujo [1,*], Andre L. A. Reis [2], Angela A. P. Correa [3], Elder Yokoyama [3], Vanderlei C. Oliveira Jr. [2], Leonardo A. F. Mendoza [4], Marcos A. C. Pacheco [5], Cleanio Luz-Lima [6], Amanda F. Santos [1], Fredy G. Osorio G. [1], Giancarlo E. Brito [7], Wagner W. R. Araujo [7], Tahir [1], Antonio C. Bruno [1] and Tommaso Del Rosso [1]**

1 Department of Physics, Pontifical Catholic University of Rio de Janeiro, Rio de Janeiro 22451–900, Brazil; afariassantos@aluno.puc-rio.br (A.F.S.); fosorio@puc-rio.br (F.G.O.G.); tahirjanqau@gmail.com (T.); acbruno@puc-rio.br (A.C.B.); tommaso@puc-rio.br (T.D.R.)

2 Department of Geophysics, National Observatory, Rio de Janeiro 20921-400, Brazil; andrelreis@on.br (A.L.A.R.); vanderlei@on.br (V.C.O.J.)

3 Institute of Geosciences, University of Brasília, Brasília 70910-900, Brazil; angela@erg.inpe.br (A.A.P.C.); eyokoyama@unb.br (E.Y.)

4 Department of Electrical Engineering, State University of Rio de Janeiro, Rio de Janeiro 20550-900, Brazil; mendonza@ele.puc-rio.br

5 Department of Electrical Engineering, Pontifical Catholic University of Rio de Janeiro, Rio de Janeiro 22451–900, Brazil; marco@ele.puc-rio.br

6 Department of Physics, Federal University of Piauí, Teresina 64049-550, PI, Brazil; cleanio@ufpi.edu.br

7 Institute of Physics, University of São Paulo, São Paulo 05508-090, Brazil; gbrito@if.usp.br (G.E.B.); wwlysses@usp.br (W.W.R.A.)

* Correspondence: jferraz@fis.puc-rio.br

Here, we describe the construction of a customized scanning magnetic microscope based on commercial Hall-effect sensors at room temperature (See Figure S1) that achieves a spatial resolution of 200 μm. Two scanning stages on the x- and y-axes of precision, consisting of two coupled actuators, control the position of the sample, and this microscope can operate inside or outside a magnetic shield. We obtained magnetic field sensitivities better than 520 $nT_{rms}/\sqrt{Hz}$ (±10 $nT_{rms}/\sqrt{Hz}$) between 1 and 10 Hz, which correspond to a magnetic momentum sensitivity of 9.20 × $10^{-10}$ $Am^2$. The standard deviation of the measurements was approximately 0.04 × $10^{-10}$ $Am^2$ (about 0.38%) for the remanent magnetization. In Figure S1 we can identify two circuit boards.

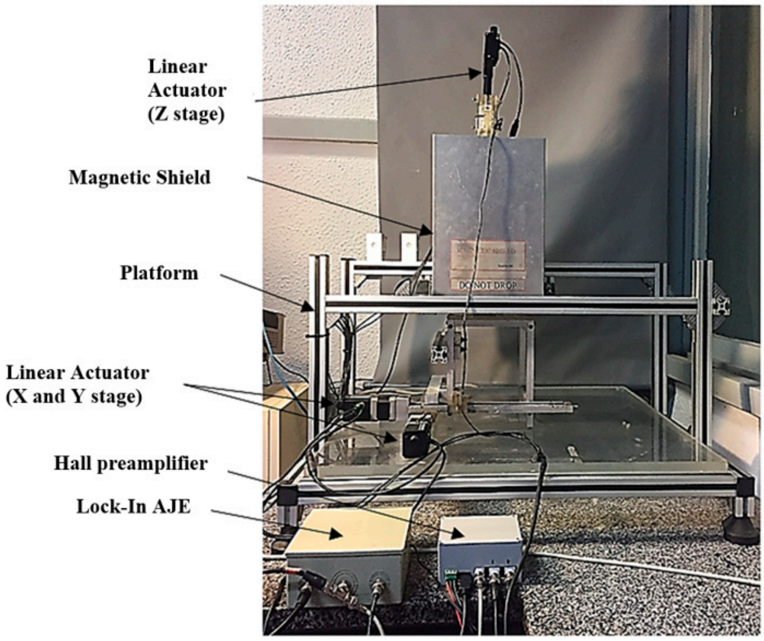


**Figure S1.** Image of the Hall microscope and its components.

We can configure the sensors by adjusting the current or voltage. After several tests, we concluded that polarization by current in the 0.5–4.5 mA range produced the best signal-to-noise ratio [1–2]. The circuit (Figure S2a) consisted of current sources and instrumentation amplifiers for the amplification. A low-noise preamplifier was built (we constructed the Lock-In that hereafter referred to using the following abbreviation: AJE amplifier) and acts as a gradiometer by electronically subtracting the two output signals. In the assembly, the current sources were based on the IC LM334 which is resistance-controlled and shows high noise and a strong temperature dependence [1–4]. Next, we redesigned the circuit, replacing the LM334 with the AMP03 that has two current sources, which are controlled by voltage, and we achieved better results. Figure S2b shows a comparison between the noise spectra of the two custom electronic devices, one with the AMP03 electronics and the fabricated Lock-In AJE amplifier and the other with the commercial Lock-In amplifier (SR560, SRS Inc.). We used an alternating current at a frequency of 1.0 kHz and a peak amplitude of 1.0 V. For comparison purposes, we added a magnetic signal at 4 Hz to the measurements.

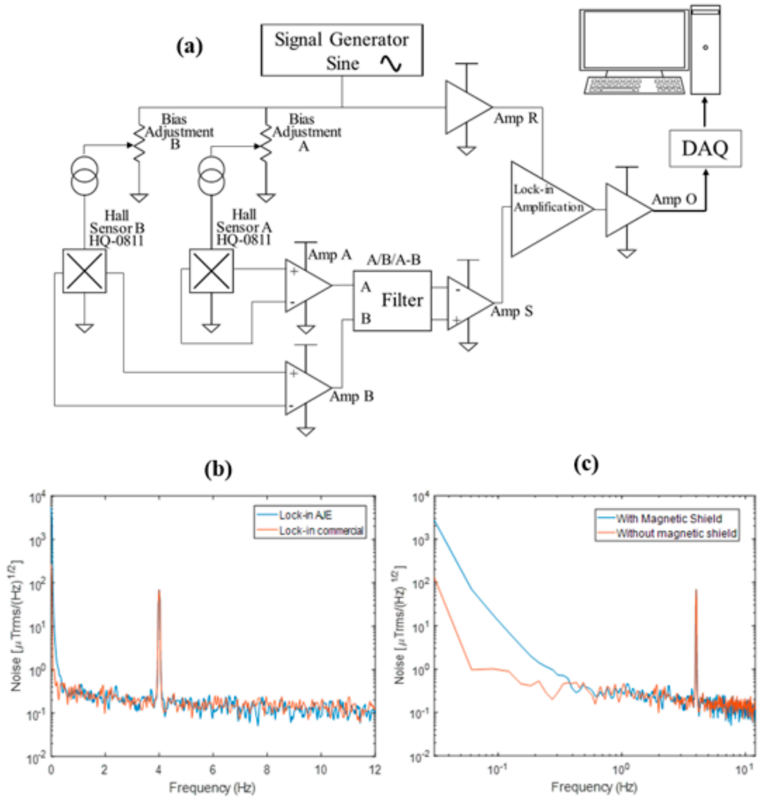


**Figure S2.** (**a**) Circuit consisting of current sources and instrumentation amplifiers for the amplification. (**b**) Noise analysis graph between the Lock-In AJE equipment and the commercial Lock-In equipment. (**c**) Noise analysis graph of the gradiometer system with and without the protection of the magnetic shield.

We can observe in Figure S2b that, under these conditions, there is virtually no difference between the two configurations (the Lock-In AJE amplifier and the commercial Lock-In equipment), and the preamplifier (the Lock-In AJE amplifier) has low operating costs. We also conducted noise tests with and without the magnetic shield (See Figure S2c) and once again observed that there is no difference in the readings of the gradiometer system with and without the protection of the magnetic shield, which is necessary for some geological samples. In the assembly of the Lock-In AJE amplifier (Figure S3), it was used low-cost but with high accuracy components found in the domestic market. It is very important to get the greatest reliability possible since the idea is to reach an accuracy to substitute larger and expensive equipment. It was used an application layout according to the technical specifications of each of the integrated circuits (ICs). The main circuit has 3 stages, first an amplification input stage managed by the AD620 instrumentation amplifier, followed by a dynamic range demodulation Lock-In handled by an IC AD630 Balanced Modulator/Demodulator and lastly filtering with an amplification output, regulated by an OP27G OPAM.

Using the help of the Proteus 8.1 software, which is specialized for electronic circuit designs, the components were assembled according to the electronic circuit diagram shown in the figure below.

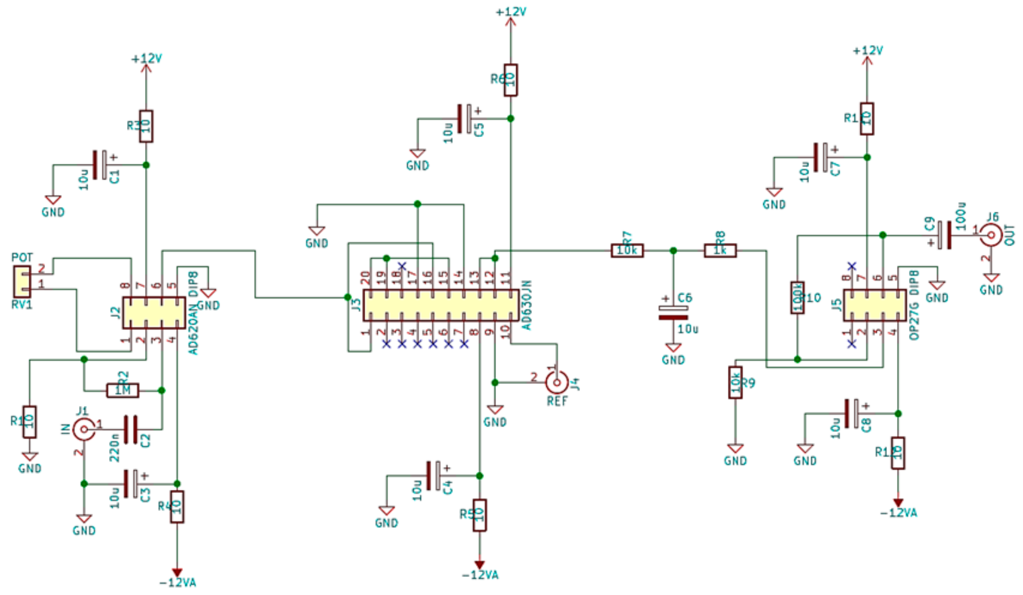


**Figure S3.** Electric circuit of a Lock-In amplifier using the AD620, AD630, and OP27 integrated circuits.

Besides the electronic components offered in the market, the operation of three ICs stood out when the objective was to assemble a specific amplification circuit with phase adjustment. The ICs selected for the manufactured Lock-In amplifier are listed below. The AD620, used as the initial amplifier, amplifies the signal applying a variable gain resistance. Since the amplifier amplifies not only the desired signal but also the signal as a whole, the noise is also amplified. Therefore, an appropriate sensitivity setting should be chosen.

The AD630 synchronous demodulator, which is an essential component in the Lock-In amplifier, mixes the real signal to form the input reference signal. The OP27G, a low-pass amplifier, filters out any noise in the modulated signal and produces the desired DC signal, which determines the filtering level. Given the technical characteristics of these ICs, one can assemble an electrical circuit.

In preliminary tests, the AD620 amplifier received the signal from the function generator, simulating the signal obtained by the magnetic probe, and amplified its voltage according to the adjustments made in the potentiometer. The operation was based simply on a noninverting operating amplifier. The circuit was powered by the +/− 15 V power source, with low-pass filters connected to each source to filter out any undesirable circulating noise. The disk capacitor worked in avoiding fluctuations in the signal when performing demodulation on the road. A reference signal, also provided by the signal generator, was introduced into the demodulator for signal mixing because this component is the signal input port to the demodulator. The synchronous demodulator (AD630) received the output signal from the input amplifier and multiplied it with the reference input. The final component of the Lock-In amplifier, the OP27 low-pass amplifier, was designed to filter all unwanted signals and produce a DC (direct current) signal that indicates the field strength. The equipment can not only be used in cutting-edge research but also serve as a teaching tool to introduce undergraduate, master's and Ph.D. students to the measurement methods and processing techniques used in scanning magnetic microscopy.

## References

1. Lima, E.A., Bruno, A.C., Carvalho, H.R., Weiss, B.P. Scanning magnetic tunnel junction microscope for high-resolution imaging of remanent magnetization fields. *Meas. Sci. Technol.* **2014**, *25*, 105401. doi: 10.1088/0957-0233/25/10/105401.

2. Pereira, J.M.B.; Pacheco, C.J.; Arenas, M.P.; Araujo, J.F.D.F.; Pereira, G.R.; Bruno, A.C. Novel scanning dc-susceptometer for characterization of heat-resistant steels with different states of aging. *J. Magn. Magn. Mater.* **2017**, *442*, 311–318. doi: 10.1016/j.jmmm.2017.07.004
3. Araujo, J.F.D.F.; Bruno, A.C.; Louro, S.R.W. Versatile magnetometer assembly for characterizing magnetic properties of nanoparticles. *Rev. Sci. Instrum.* 2015, 85, 105103–105107. doi: 10.1063/1.4931989
4. Reis, A.L.A.; Oliveira Jr, V.C.; Yokoyama, E.; Bruno, A.C.; Pereira, J.M.B. Estimating the magnetization distribution within rectangular rock samples. *Geochem. Geophys. Geosyst.* **2016**, *17*, 3350–3374, doi:10.1002/2016GC006329.

© 2019 by the authors. Submitted for possible open access publication under the terms and conditions of the Creative Commons Attribution (CC BY) license (http://creativecommons.org/licenses/by/4.0/).
